# Sorghum, or Chinese Sugar Cane

**Published:** 1858-02

**Authors:** 


					﻿Sorghum Saccharatum.—'We are indebted to the politeness of Dr. Isaac
Hays, for a detailed account of the experiments of Joseph S. Lovering on
the Chinese sorghum, which have been fallowed by such successful results.
We have not ourselves supposed that the sorghum would never be made
into sugar; but in an article in the January number, merely quoted the
statement of a chemist connected with one of the Southern sugar manufac-
tories, that it could never be made to granulate. We extract a synopsis of
the conclusions arrived at by Mr. Lovering, from his pamphlet containing an
account of his experiments.	r.
1st. That it is obvious that there is a culminating point in the develop-
ment of the sugar in the cane, which is the best time for sugar making.
This point or season I consider to be, when most if not all the seeds are ripe,
and after several frosts, say when the temperature falls to 25° or 30° F.
2d. That frost, or even hard freezing, does not injure the juice nor the
sugar, but that warm Indian summer weather, after the frost and hard freez-
ing, does injure them very materially, and reduces both quantity and quality.
3d. That if the cane is cut and housed, or shocked in the field when in
its most favorable condition, it will probably keep nnchanged for a long
time.
4th. That when the juice is obtained the process should proceed continu-
ously and without delay.
5th. That the clarification should be as perfect as possible by the time the
density reaches 15° Beaume, the syrup having the appearance of good
brandy.
6th. That although eggs were used in these small expeiiments, on ac-
count of their convenience, bullock’s blood, if to be had, is equally good, and
the milk of lime alone will answer the purpose; in the latter case, however,
more constant and prolonged skimming will be required to produce a per-
fect clarification, which is highly important.
7th. That the concentration, or boiling down, after clarification, should be
as rapid as possible without scorching—shallow evaporators being the best.
With these conditions secured, it is about as easy to make good sugar
from the Chinese cane as to make a pot of good mush, and much easier than
to make a kettle of good apple butter.
Dr. Austin Flint. — We have the honor and the above mentioned gen-
tleman labors under the disadvantage of bearing this name, and inasmuch
as we occasionally appear before the medical public, articles which emanate
from our individual pen are sometimes attributed to the senior. We notice
this mistake in a kind notice in the American Journal of Dental Science, of
a paper on the capillary circulation, which appeared in the July, 1857, No.
of the American Journal of Med. Sciences, of which Dr. F. sen., would, per-
haps, be glad to say that he is entirely innocent. As this paper was here
kindly mentioned, we feel it our duty to exonerate Prof. Flint from its
authorship, though under other circumstances we would not, perhaps, be
unwilling that he should father some of our iniquities. In future, if any of
our editorial confreres are pleased with an effort of ours, they will be kind
enough to remember the <7r., if otherwise, it is not so material.	f.
A Practical Treatise on the Diseases of Children. By J. Forsyth Meigs
M. D., Fellow of the College of Physicians of Philadelphia; Member of
the American Philosophical Society, and of the Academy of Natural Sci-
ences of Philadelphia. Third Edition, carefully Revised. Philadelphia:
Lindsay & Blakiston. 1858.
This admirable text-book on the diseases of children, has now become a
standard work. The labors of the author have added much entirely new
matter to the first edition, which formed one of a series entitled “The Medi-
cal Practitioner’s and Student’s Library.” The third edition has been care-
fully revised, and much new matter incorporated into the text, though the
author has been prevented from making additions of chapters on subjects
not touched upon, which he had proposed to do in the present edition. We
hope that this promise will be fulfilled, however, in another edition. f.
American Medical Monthly. — The proprietors of this Journal have en-
larged the volume by an addition of sixteen pages to each number. We are
glad to see this evidence of prosperity, and can bear testimony that it is well
deserved. The Monthly has always been conducted with high-minded spirit
and candor on the part of the editors, and its pages have been enriched by
many valuable contributions to science.	f.
Editorial Change. — With the last number of the twelfth volume of the
Charleston Medical Journal, Dr. C. Happolt retires from its editorial man-
agement and proprietorship. Dr. Happolt retires with our sincere wishes
for his future welfare and our respect for the able manner in which he has
conducted his journal. The new editor, Dr. J. Dickson Bruns, is already
favorably known to the profession by some important and interesting contri-
butions, and will no doubt sustain himself well in his new position. f.
The North American Medico- Chirurgical Review. — The size of this
sterling periodical has been enlarged with the cammencement of the second
volume, making now one hundred and ninety-two pages to each number,
this journal was commenced in Philadelphia on the first of January, 1857,
by Profs. Gross and Richardson, the former editors of the Louisville Review;
the sound and able articles and reviews which have appeared in its pages
for the past year have made it one of the most agreeable and profitable of
our exchanges, and have rendered its success sufficiently flattering to induce
the publishers to enlarge it to its present dimensions. The appearance which
it now presents in point of typography and style is highly creditable, and
the editorial management is such as to make it rank with the best Journals
of any country.	f.
Consolidation. — The Western Lancet and the Cincinnati Medical Ob-
server, have been consolidated under the name of the Cincinnati Lancet &
Observer. Some of the circumstances which induced this change are men-
tioned in the last number of the Lancet. The want of a sufficiently ener-
getic corps of colaborators, and the desire to diminish the number of of rival
factions and interests, and unite the talent of her writers, are mentioned as
the principal causes which have made this step advisable. The consolidated
number looks well.	f.
A New Medical School at Nashville. — We learn that the brilliant suc-
cess of the medical department of the University has prompted a new enter-
prise in the way of a Medical School at Nashville. We understand that it
is to be under the patronage of the Methodist denomination.— Cincinnati
Lancet A Observer.
The University of London and Homoeopathy. — At a late examination
for the degree of M. B. of the University of London, a candidate, who was
known to profess and practice homoeopathy, was rejected.—lb.
				

